# Geospatial mapping and resource utilization tool in support of a national smoke-free public housing rule

**DOI:** 10.1186/s13104-019-4815-x

**Published:** 2019-11-25

**Authors:** Sonia Tetlow, Brian Gurbaxani, Corinne Graffunder, Connor Owen, Diem Tran, Jiali Zhao, Jose A. Rodriguez, Aaron Ahn, Kristie Choe, Vishal Mummigatti, Divya Vedula, Kathleen Hayes, Miriam Kelly, Simon McNabb, Julie Swann

**Affiliations:** 1 Office on Smoking and Health, National Center for Chronic Disease Prevention and Health Promotion, Centers for Disease Control and Prevention (CDC), 4770 Buford Highway, Atlanta, GA 30341 USA; 2 Office of Science, Deputy Director for Public Health Science and Surveillance, CDC, Atlanta, GA USA; 3 School of Industrial and Systems Engineering, Georgia Institute of Technology, Atlanta, GA USA; 4 Department of Industrial and Systems Engineering, North Carolina State University, Raleigh, NC USA

**Keywords:** Tobacco, Smoking cessation, Smoke-free policy, Secondhand smoke, Public housing, Public health, Multiunit housing, Geographic information system, Geospatial

## Abstract

**Objective:**

To advance public health support for the U.S. Department of Housing and Urban Development’s smoke-free rule, the Centers for Disease Control and Prevention collaborated with the Georgia Institute of Technology to develop a geospatial mapping tool. The objective was to create a tool state and local public health agencies could use to tailor smoke-free educational materials and cessation interventions for specific public housing development resident populations.

**Results:**

The resulting “Extinguish Tool” includes an interactive map of U.S. public housing developments (PHDs) and healthcare facilities that provides detailed information on individual PHDs, their proximity to existing healthcare facilities, and the demographic characteristics of residents. The tool also estimates the number of PHD residents who smoke cigarettes and calculates crude estimates of the potential economic benefits of providing cessation interventions to these residents. The geospatial mapping tool project serves as an example of a collaborative and innovative public health approach to protecting the health and well-being of the nation’s two million public housing residents, including 760,000 children, from the harms of tobacco smoking and secondhand smoke exposure in the places where they live, play, and gather.

## Introduction

Approximately 480,000 Americans die from cigarette smoking each year, including 41,000 from secondhand smoke (SHS) exposure [1]. Smoking in the U.S. also results in over $300 billion annually in direct medical care costs and lost productivity due to premature death and exposure to SHS [1, 2]. While the percentage of U.S. adults who smoke cigarettes declined to 14% in 2017 from 20.9% in 2005 [3], smoking among certain segments of the population remains disproportionately high [4]. One-third of adults living in public housing currently smoke cigarettes [5]. Consequently, the estimated 2 million individuals who live in U.S. public housing, including 760,000 children, are at risk of adverse health outcomes associated with smoking and exposure to SHS [5].

On February 3, 2017, the U.S. Department of Housing and Urban Development (HUD), which oversees public housing, enacted a rule requiring agencies that administer public housing to implement a smoke-free policy within 18 months [6]. As part of its multifaceted efforts to support implementation of HUD’s rule, the Centers for Disease Control and Prevention’s (CDC) Office on Smoking and Health collaborated with CDC’s Office of Science and a team of senior design students from the Georgia Institute of Technology (Georgia Tech) to develop an interactive geographic information system (GIS) mapping tool. The objective was to create a tool state and local public health agencies could use to tailor smoke-free educational materials and cessation interventions for specific public housing development (PHD) resident populations. The resulting “Extinguish Tool” combines GIS mapping, small area estimation, and economic benefit estimation into one online resource that provides information previously unavailable from existing mapping tools, including estimates of the number of residents who smoke in each PHD [7].

## Main text

The Extinguish Tool is available for use online [8]. The homepage map was scripted in Leaflet, an open-source JavaScript library. The map template upon which the public health and public housing data is displayed was provided by the location data platform, Mapbox. All other visualizations were created using an open-source JavaScript library, D3.js. All source coding is available for the user to see using right-click “View Source” or from Github [9].

The Extinguish Tool mapping component draws on location information from seven publicly available federal datasets. The HUD PHD dataset provides the locations of each U.S. public housing development, defined by the address of the building within each development with the largest number of units [10]. Additional datasets include Federally Qualified Health Centers, Healthcare Facilities of the Indian Health Service, Home Health Services, Hospitals, and Medical Centers from the U.S. Department of Health & Human Services and a dataset of Veterans Health Administration Facilities from the U.S. Department of Veterans Affairs [11, 12]. These datasets are connected to the tool via an application programming interface (API), which automatically transmits any changes to the datasets made by the federal agencies to the tool.

The HUD PHD dataset also provides information on resident demographic characteristics, including sex, age, race/ethnicity, income level, disability status, and marital status. As a housing dataset, it does not include information related to cigarette smoking status. Thus, the tool uses a two-part regression and simulation model (small-area estimation) to generate estimates of the number of residents who smoke cigarettes in each PHD [13, 14]. The regression model predicts individual cigarette smoking status, and the simulation model uses those predictions to generate estimates of the number of residents who smoke cigarettes in each PHD.

### Methods

Variables from the 2015 Behavioral Risk Factor Surveillance System (BRFSS), a national dataset of state-level survey data collected by all 50 U.S. states and participating territories, that aligned with resident characteristics variables in the HUD PHD dataset were used in the regression model [15, 16]. Two HUD PHD variables included multiple resident characteristics in a single variable. In order to match them, similar individual data points from the BRFSS dataset were combined into categorical variables for the regression model. A proxy for disability status was created using the BRFSS variables for employment status where the value “unable to work” was indicated and for blindness where the value “blind or serious difficulty seeing, even when wearing glasses” was indicated. Additional independent variables not related to the HUD PHD dataset included information on residential environment from the BRFSS dataset and state cigarette excise tax rates [17] (Table 1).

**Table 1:** Regression model variables used to predict cigarette smoking status

Variable type	Variable categories
State tax rate for a pack of 20 cigarettes	Not applicable
Residential environment	City
	Suburb
	Rural
Race/ethnicity	White
	Black/African American
	American Indian/Alaskan Native
	Asian and Native Hawaiian/Pacific Islander
	Hispanic
	Other/multiracial
Age	18–24
	25–49
	50–59
	60–79
	80 or above
Reported household income	< $10 k
	$10,001–$15 k
	$15,001–$20 k
	≥ $20,001
A variable that combines gender, marital status, and presence of children (to match the HUD dataset variable)	Two spouses/married/with children
	Two spouses/married/no children
	Female/not married/with children
	Female/not married/no children
	Male/not married/with children
	Male/not married/no children
A variable that combines age and disability status (to match the HUD dataset variable)	Age < 65 with disability
	Age < 65 no disability
	Age > 65 with disability
	Age > 65 no disability

**Fig. 1:**
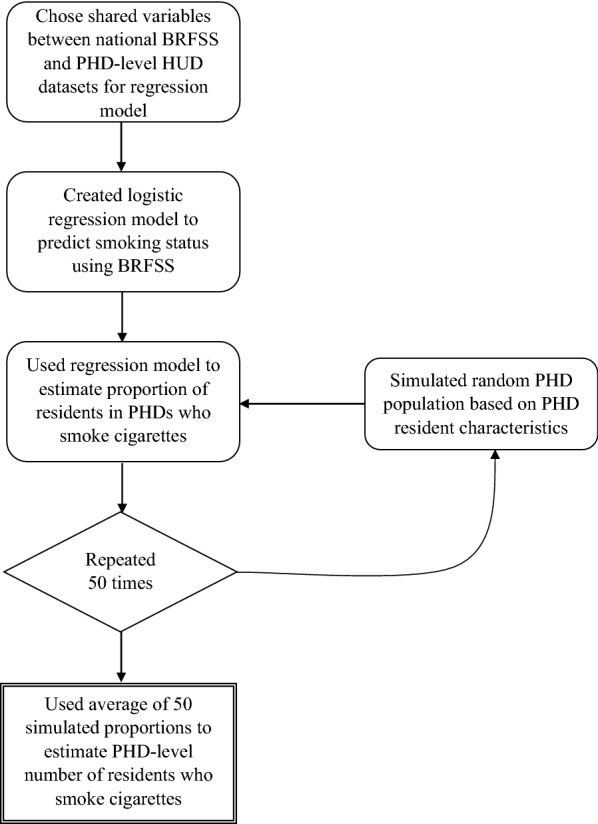
Small area estimation for PHD residents who smoke cigarettes

A logistic regression model was run on R statistical software to predict cigarette smoking status (Eq. 1):

1 $$l_{sm} = \beta_{tax} + \beta_{1} x_{res} + \beta_{2} x_{race} + \beta_{3} x_{age} + \beta_{4} x_{\$ } + \beta_{5} x_{GenMaCh} + \beta_{6} x_{dis} ,$$

where l_sm_ is the log odds of smoking status, β_tax_ is the state excise tax rate on cigarettes, x_res_ is the categorical variable for residence environment, x_race_ is the categorical variable for race, x_age_ is the categorical variable for age, x_$_ is the categorical variable for household income, x_GenMaCh_ is the combined categorical variable for gender/marital/child at home status, x_dis_ is the combined categorical variable for disability status, and β_1–6_ are the regression coefficients—all as shown in Table 1. The logistic regression equation shown was fit to the entire nationwide dataset and cross validated (75% of the data used to train, 25% to test, 100 times). All of the regression coefficients were significant with a $$p < 10^{ - 10}$$ (in most cases $$p < 2 \cdot 10^{ - 16}$$). A threshold for the dependent variable (l_sm_) of 0.33 was chosen so that, when the equation itself was applied to the data, the national estimates of cigarette smoking prevalence among HUD residents was matched [5]. This also resulted in a small false positive rate of about 5%. However, when the regression equation was applied to all of the individual state datasets in the BRFSS, so the sensitivity and specificity were allowed to vary, the AUC (area under the curve) was only fair at 0.69, indicating moderate predictive power. To mitigate some of this variation in predictive power, estimates for a given PHD were computed by averaging 50 applications of the regression equation to bootstrap samples from the PHD (Fig. 1). This calculation enabled the small area estimation of smoking status.

The simulation model used the information about actual PHD residents available at the aggregate level from HUD’s PHD dataset and the regression coefficients to generate simulated PHD residents at an individual level and predict their cigarette smoking status. County level estimates of PHD residents who smoke cigarettes reflect the total number of residents estimated to smoke among the total public housing population residing in a given county.

The estimates of PHD residents who smoke were used to calculate crude estimates of the potential economic benefits of providing cessation interventions to those residents. Each estimate was based on the intervention’s estimated effectiveness and the before and after number of PHD residents predicted to smoke according to the regression and simulation models. Smoking-related medical costs were considered from a societal perspective. The return on investment (ROI) estimates thus were calculated by subtracting the estimated annual costs with the intervention from the estimated annual costs without the intervention and dividing by the annual cost of the intervention.

The ROI estimates (Eq. 2) were derived as follows:

2 $${\text{ROI}} = \frac{{\left[ {{\text{Est cost with no intervention}} - {\text{Est cost with intervention}} } \right]}}{\text{Est cost of intervention}}$$

An ROI > 1 would indicate that the estimated smoking-related medical costs averted were predicted to be greater than the cost of the cessation intervention. When the model was tested on two PHDs in Georgia and New York, the ROI estimates generated were positive, showing that costs averted were greater for all interventions in both states.

The cross-sector cost savings estimates were based on the potential reduction in costs relevant to the public housing system due to predicted reductions in the number of PHD residents who smoke after successful cessation. These included costs associated with evictions, turnover of smoking units, and smoking-related fires [18]. Estimated cost savings were calculated by subtracting the estimated annual costs with the intervention from the estimated annual costs without the intervention. The results provide crude estimates of the potential cost savings to the public housing sector that could be realized in addition to the positive ROI estimates from averted medical costs.

### Result

**Fig. 2:**
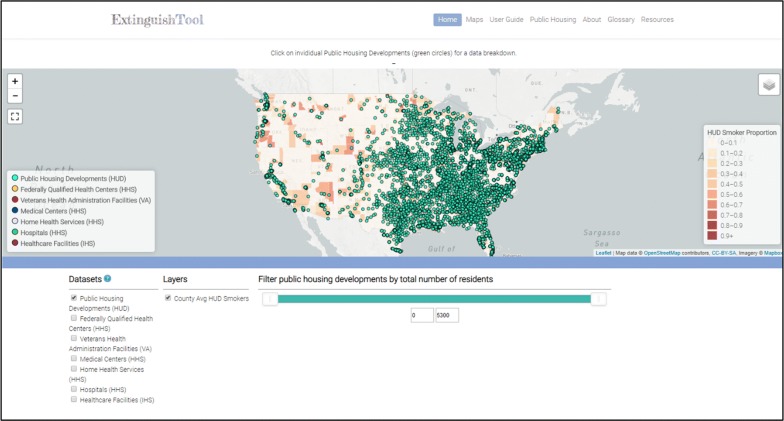
The Extinguish Tool Homepage. The image provides a screen shot of the Extinguish Tool homepage, which auto-populates with a heatmap depicting the estimated proportion of public housing development residents who smoke cigarettes on a county level and green indicators for each public housing development

The Extinguish Tool launched on a Georgia Tech website in April 2017. The homepage displays the interactive mapping tool that initializes with indicators for the locations of PHDs and a heatmap depicting the estimated proportion of PHD residents who smoke on a county level. The PHDs shown on the map can be filtered by total number of residents in order to examine PHD locations by population size. Indicators for the healthcare facilities whose datasets are listed under the map may be activated or deactivated to customize the types of facilities displayed. Users may view PHD and healthcare facility locations at a local or national scale (Fig. 2).

When a user hovers over an indicator for a PHD or healthcare facility, the name will appear in a light box over the map. When a user clicks on an indicator for a PHD, the tool engages and two datasets are queried to provide the PHD-specific information displayed below the map. The first is the HUD PHD dataset, which is dynamic and connected via an API, and provides the PHD name, address, contact information, and aggregate resident demographic characteristics. The second dataset, which is static and was derived through the regression and simulation model, provides an estimate of the number of PHD residents who smoke cigarettes in the development, and the estimated economic benefits of providing those residents with cessation interventions.

### Discussion

The 18 months between when HUD enacted its smoke-free rule and the deadline for implementation presented a unique opportunity for public health agencies to determine supportive strategies in circumstances where the intervention, timeline, and intended population all were known in advance. The Extinguish Tool supplemented CDC’s multifaceted efforts to support implementation of HUD’s smoke-free rule. It demonstrated the innovative integration of GIS technology with regression and simulation modeling and the potential for using applied research to create a practical tool for those working in the field. The Extinguish tool provided public health professionals with a quick and easy way to access previously unavailable information, including the locations of local PHDs, the characteristics of residents living in each PHD, and estimates of the number of residents who smoke cigarettes, and to assess PHD proximity to healthcare facilities where residents could access cessation treatment. This information could be used to tailor smoke-free education materials and cessation interventions for specific resident populations. Tailoring health communication materials for specific audiences can improve their effectiveness [19]. The Extinguish Tool provides state and local stakeholders with a practical resource to support their efforts to protect the health and well-being of the nation’s two million public housing residents, including 760,000 children. Further research is needed to evaluate the utility of the tool in practice and how it may be refined or improved.

## Limitations

There are some limitations that should be considered when interpreting the information generated by the Extinguish Tool. First, the number of PHD residents who smoke and the economic benefits are crude estimates resulting from the regression and simulation model. Second, the model itself is limited by the HUD PHD Dataset resident characteristics variables and does not include some indicators associated with a higher risk of tobacco use, such as level of education or previous attempts to quit smoking. Third, the economic benefit estimates are rough point-in-time estimates that do not take into account the time horizon of implementation and successful cessation in the short-term or the potential impact on cost and benefits in the long-term. Additionally, published estimates of cessation intervention efficacy and smoking-related costs were used in the ROI model and assumptions applied globally to all PHD resident populations. Finally, the tool only provides estimates on cigarette smoking and not all forms of combustible tobacco smoking (e.g. cigars, hookah, and pipes) or use of other tobacco products that individual public housing authorities may choose to voluntarily include in their policies beyond those specified in the final rule (e.g. electronic cigarettes).

## Data Availability

The datasets used in the tool are publicly available from: U.S. Department of Housing and Urban Development (https://hudgis-hud.opendata.arcgis.com/datasets/public-housing-developments); US Department of Health & Human Services, http://open-fedmaps.opendata.arcgis.com/datasets?q=Department%20of%20Health%20&%20Human%20Services&sort_by=relevance; U.S. Department of Veterans Affairs, https://www.data.va.gov/; and Centers for Disease Control and Prevention, https://www.cdc.gov/brfss/. The Extinguish Tool’s technical guide and ROI model are available on the tool’s “User Guide” webpage: https://nchh.org/extinguish-tool/userGuide.html.

## Abbreviations

API: application programming interface
BRFSS: Behavioral Risk Factor Surveillance System
CDC: Centers for Disease Control and Prevention
GEORGIA TECH: Georgia Institute of Technology
GIS: Geographic Information System
HUD: U.S. Department of Housing and Urban Development
PHD: public housing development
ROI: return on investment
SHS: secondhand smoke
